# Efficacy of Shexiang Baoxin Pills for the treatment of unstable angina pectoris: Protocol of systemic review and meta-analysis

**DOI:** 10.1097/MD.0000000000017119

**Published:** 2019-09-13

**Authors:** Huadong Chen, Qianqian Dong, Xiang Zheng

**Affiliations:** Department of Pharmacy, Dongyang People's Hospital, Dongyang, Zhejiang Province, China.

**Keywords:** complementary medicine, evidence-based medicine, Shexiang Baoxin Pill, traditional Chinese medicine, unstable angina pectoris

## Abstract

**Background:**

Shexiang Baoxin Pills (SBP) is widely used for the treatment of unstable angina pectoris (UAP) in China. However, the clinical evidence on the efficacy of SBP for the treatment of UAP is not well concluded.

**Methods:**

Seven electronic databases will be searched for eligible studies: MEDLINE, EMBASE, The Cochrane Library, Wanfang database, Chinese National Knowledge Infrastructure database, VIP database, and Chinese Biological and Medicine database. Data of included studies will be extracted, and quality will be evaluated. Data synthesis will be performed using RevMan software. Subgroup analysis and sensitivity analysis will also be carried out. Publication bias will be evaluated using funnel plot if included studies are sufficient.

**Results:**

This systemic review and meta-analysis will provide synthesized result of clinical efficacy of SBP for the treatment of UAP.

**Conclusions:**

This systemic review and meta-analysis will provide high-quality evidence on the clinical efficacy of SBP for the treatment of UAP.

**Registration:**

PEROSPERO CRD42019124668

## 1 Introduction

Unstable angina pectoris (UAP) belongs to a spectrum of ischemic heart disease that is called acute coronary syndrome.^[1]^ Recent real-world data demonstrated a relative increase in the incidence of UAP by 13% among patients admitted to Swedish coronary care units.^[2]^ UAP patients tended to be younger and had a higher prevalence of most cardiovascular risk factors and a greater burden of coronary artery disease than those with non-ST elevation myocardial infarction.^[3]^ The pharmacological therapies of UAP can be divided into 2 categories: anti-ischemic and anti-platelet/anti-coagulation. Anti-ischemic therapies include nitroglycerin, beta blockers, and angiotensin-converting enzyme inhibitors. Anti-platelet or anti-coagulation therapies include aspirin, P2Y12 receptor inhibitors, heparin, and so on.^[4]^ Improvements in primary prevention and acute management strategies have led to a reduction in in-hospital mortality; however, the incidence continues to be stable.^[5]^ Moreover, the medical expenditure among inpatients with UAP is quite high.^[6]^ New therapies is still need for UAP.

Complementary medicine, especially traditional Chinese medicine (TCM), plays an important role in treating UAP in China. Several TCM prescriptions are proved to have good clinical effect for the treatment of UAP, and Shexiang Baoxin Pills (SBP) is one of the most promising prescription.^[7–10]^ SBP consists of 7 herbal medicine, namely, musk (*Moschus*, She Xiang), ginseng root (*Radix Ginseng*, Ren Shen), cow bezoar (*Calculus bovis*, Niu Huang), storax (*Styrax*, Su He Xiang), cassia bark (*Cortex Cinnamomi*, Rou Gui), toad venom (*Venenum Bufonis*, Chan Su), and borneol (*Borneolum syntheticum*, Bing Pian).^[3]^ The major active components of SBP includes ginsenosides, bile acids, bufadienolides, and volatiles.^[11]^ Modern pharmacological researches have revealed that SBP and its active components display pleiotropic roles in protecting the cardiovascular system, as seen by the promotion of angiogenesis, amelioration of inflammation, improvement of endothelium dysfunction, and so on, which may be important for its efficacy on UAP.^[12–15]^ SBP is proved to be beneficial as an adjuvant therapy in many cardiovascular diseases, such as non-ST elevation acute coronary syndrome and heart failure.^[16–18]^ However, the clinical evidence on the efficacy of SBP for the treatment of UAP is not well concluded. Here we propose this systemic and meta-analysis protocol to evaluate the clinical efficacy of SBP for the treatment of UAP.

## 2 Methods

This protocol is registered in PEROSPERO (CRD42019124668) and written following the Preferred Reporting Items for Systemic Review and Meta-analysis Protocol (PRISMA-P).^[19]^ Ethical approval is not needed because this research only involves published data.

### 2.1 Eligibility criteria

#### 2.1.1 Type of study

Only randomized clinical trials (RCTs) would be included. Studies which have not claimed as RCT but meet the criteria of RCT will also be included.

#### 2.1.2 Participants

Patients with UAP diagnosed using any recognized diagnostic criteria. There is no restriction regarding the severity or length of the UAP. Patients with other types of angina pectoris or unclassified angina pectoris will be excluded.

#### 2.1.3 Interventions

UAP patients treated with SBP alone or combination of SBP and other drugs. There is no restriction of the dose of SBP or the types of combined drugs.

#### 2.1.4 Comparison

UAP patients treated with drugs other than SBP. Patients not treated or treated with placebo will also be included. Patients treated with other physical complementary therapies such as Qigong and acupuncture will also be included.

#### 2.1.5 Publication data and language

Studies published before May 1, 2019 will be sought. There is no restriction regarding the publication language.

### 2.2 Information source

The following electronic databases will be searched as information source: MEDLINE, EMBASE, The Cochrane Library, Wanfang database, Chinese National Knowledge Infrastructure (CNKI) database, VIP database, and Chinese Biological and Medicine (CBM) database.

### 2.3 Search strategy

The mentioned electronic databases will be searched using a combination of following search items: Shexiang Baoxin; She Xiang; musk; *Moschus*; unstable angina pectoris; acute coronary syndrome/disease; ischemic heart disease; and myocardial infarction. The combination of search items and search strategy will be adjusted to suit the specific database. Relative literatures such as references of included studies will be searched manually. The literature search will be carried out by 2 reviewers (Q.D. and X.Z.) independently. The search results will be crosschecked, and any discrepancies will be solved by discussing with a third reviewer (H.C.).

### 2.4 Study selection and data extraction

The searched studies will be managed using an electronic reference managing software, Endnote. Two reviewers (Q.D. and X.Z.) will review the searched studies. Title and abstracts of studies retrieved will be screened by reviewers independently to identify studies that potentially meet the inclusion criteria. The full text of these potentially eligible studies will be retrieved and further assessed. Reviewers will select studies according to the eligibility criteria independently. Any discrepancies between the 2 reviewers will be solved with a third reviewer (H.C.).

An Excel electronic table will be applied to manage the extracted data. The following items of eligible studies will be extracted: author and published year, study population and participant demographics, study religion and duration, baseline characteristics, study design, details of intervention and control conditions, outcomes and times of measurement, and information for assessment of the risk of bias.

### 2.5 Outcomes

#### 2.5.1 Primary outcome

Clinical effective rate is the primary outcome of this research. Clinical effective is defined by the original study. If the original study has not defined, clinical effective will be defined as above 50% reduction in frequency of angina attacks and weekly frequency of angina attacks reduction.

#### 2.5.2 Secondary outcome

(1) Frequency and duration of angina attack; (2) Electrocardiogram (ECG) improvements.

### 2.6 Risk of bias in individual study

The risk of bias will be assessed using the Cochrane risk of bias tool. The assessment will be carried out by 2 reviewers (Q.D. and X.Z.) independently, and any discrepancies will be solved by discussing with a third reviewer (H.C.).

### 2.7 Data synthesis

The extracted data will be reviewer for feasibility of data synthesis. Qualitative presentation will be carried out if included studies are not enough. Data synthesis will be run with Review Manager 5.3 software (Copenhagen: The Nordic Cochrane Centre, The Cochrane Collaboration, 2014). The odds ratio and 95% confidential interval will be calculated for categorical outcomes. The mean difference and 95% confidential interval will be calculated for continuous outcomes. Heterogeneity among studies will be evaluated using the *I*^2^ test before data synthesis, and *I*^2^ > 50% is defined to indicate significant heterogeneity. The Mantel–Haenszel fixed effect model will be used when no significant heterogeneity exists among studies; otherwise, a random model will be used.

### 2.8 Subgroup analysis

Subgroup analysis will be run based on the following items if included studies are sufficient: combined drugs; dose of SBP; and severity of UAP.

### 2.9 Sensitivity analysis and publication bias

The sensitivity analysis will be carried out using 2 methods. One is the leaving-one-out method. Briefly, the synthesized outcome will be calculated by excluding studies one by one. Another method is changing the synthesized model from Mantel–Haenszel fixed effect to random effect or random effect to Mantel–Haenszel fixed effect. The robustness of synthesized result will be evaluated.

Publication bias will be investigated using funnel plot if included studies are more than 10.

### 2.10 Summary

The results of the primary outcome will be summarized using the Grading of Recommendations Assessment, Development, and Evaluation approach.

### 2.11 Dissemination plan

The finished systemic review and meta-analysis will be submitted to peer-reviewed journal and presented at international conferences.

## 3 Discussion

This protocol presents the methodology of systemic review and meta-analysis evaluating the efficacy of SBP for the treatment of UAP. This systemic review and meta-analysis will be run and reported according to the PRISMA guideline, and the efficacy of SBP for the treatment of UAP will be concluded for the first time. High-quality evidence regarding this issue will be provided. The result of this research will be helpful to physicians when choosing drugs for UAP patients. The major limitation is that the credibility of the finding will be affected by the quality of included studies.

## Author contributions

**Conceptualization:** Huadong Chen, Qianqian Dong.

**Funding acquisition:** Huadong Chen.

**Methodology:** Huadong Chen, Qianqian Dong, Xiang Zheng.

**Supervision:** Huadong Chen.

**Writing – original draft:** Qianqian Dong, Xiang Zheng.

**Writing – review & editing:** Huadong Chen, Qianqian Dong, Xiang Zheng.

Huadong Chen orcid: 0000-0002-3100-6494.
